# Brain MRI and neurocognitive characteristics of children and adolescents living with HIV

**DOI:** 10.1080/09297049.2025.2517150

**Published:** 2025-06-23

**Authors:** Manuela Martín-Bejarano García, Charlotte Jackson, Liubov Okhonskaia, Evgeny Voronin, Vladimir Rozenberg, Maria Alekseevna Titova, Tatyana Kovalenko, Catherine Wedderburn, Intira Jeannie Collins, Siobhan Crichton, Carlos Velo Higueras, Elena Salvador, María Isabel González-Tomé, Anna Turkova, José Tomás Ramos-Amador, Ana Martinez de Aragón

**Affiliations:** a Foundation for Biomedical Research of the Hospital Universitario Clinico San Carlos, Madrid, Spain; b MRC Clinical Trials Unit, University College London, London, UK; c Republican Clinical Hospital for Infectious Diseases, St Petersburg, Russian Federation; d Children’s Consultative and Diagnostic Center, Federal State Budgetary Educational Institution of Higher Education “ North-Western State Medical University named after I I. Mechnikov”, St Petersburg, Russian Federation; e Department of Paediatrics & Child Health, University of Cape Town, Cape Town, South Africa; f Neuroscience Institute, University of Cape Town, Cape Town, South Africa; g Foundation for Biomedical Research of the Hospital Universitario 12 de Octubre, Madrid, Spain; h Radiology Department, Hospital Universitario 12 de Octubre institution, Madrid, Spain; i Paediatric Infectious Diseases Department, Hospital Universitario 12 de Octubre, Madrid, Spain; j Department of Pediatrics, Hospital Clínico San Carlos, Fundación de Investigación Biomédica Hospital Clínico San Carlos (IdISSC), Madrid, Spain; k Department of Paediatrics, Instituto de Investigación Sanitaria 12 de Octubre, Universidad Complutense Madrid (UCM), Madrid, Spain; l Centro de Investigación Biomédica en Red en Enfermedades Infecciosas (CIBERINFEC), Madrid, Spain

**Keywords:** HIV, neuroimaging, cognition, Russia, child

## Abstract

Despite improved outcomes with modern antiretroviral therapy (ART), children living with HIV (CLWHIV) may still face significant cognitive deficits. There are no published studies of the neuro-cognitive and neuroimaging status of CLWHIV in Eastern Europe. This was a cross-sectional study in a pediatric HIV referral center in St Petersburg, Russian Federation. Thirty-seven CLWHIV under-went brain magnetic resonance imaging (MRI) and completed the Wechsler Intelligence Scale for Children, third edition (WISC-III) as part of routine care in 2013–15. WISC-III scores were summarized for full-scale intelligence quotient (FSIQ), performance IQ (PIQ) and performance IQ (PIQ) (all with population mean 100), and for subtests of these scores. Factors associated with MRI abnormalities were assessed using logistic regression. Median [IQR] age at the time of the earlier assessment (either MRI or WISC-III) was 10.5 [8.7–11.9] years, 54% were female, 35/ 37 (95%) had initiated ART at a median age of 57 [27–93] months. Median WISC-III scores were within the average range: 99 [IQR 91–111, range 62–123] for FSIQ, 97 [IQR 85–111, range 67–129] for VIQ and 101 [IQR 94–106, range 62–129] for PIQ. Thirty-four children (92%) scored below average on at least one subtest score. Of 32 children who underwent MRI, 15 (47%) had at least one abnormality. Older age at ART start was associated with MRI abnormalities (OR 1.37 (95% CI 1.01–1.86), *p* = 0.05 - per year increase). Although median group indexes were within the average range, a high percentage of CLWHIV underperformed in at least one subtest and might benefit from supportive interventions.

Neurocognitive impairment has been described to occur more frequently in children living with HIV (CLWHIV) compared to HIV-negative peers (Martín-Bejarano et al., 2021; Nichols, 2022; Phillips et al., 2016; Valcour et al., 2011; Wachsler-Felder & Golden, 2002). Executive functions, including working memory, may be among the most affected cognitive domains in CLWHIV (Phillips et al., 2016). Poorer cognitive function has been reported amongst children with more severe HIV disease, while early antiretroviral therapy (ART) initiation and virological suppression during infancy or early childhood are associated with better neurocognitive outcomes (Nichols, 2022).

Brain magnetic resonance imaging (MRI) is used in clinical practice to support diagnosis and monitoring of central nervous system (CNS) abnormalities in CLWHIV. White matter (WM) lesions are the most commonly reported findings, with basal ganglia calcification and cortical atrophy also described. MRI aids early detection of HIV-related CNS conditions through pattern recognition, even without advanced imaging tools. Previous work has shown that conditions such as HIV encephalopathy typically present with diffuse WM changes and cerebral atrophy on conventional MRI (Sakai et al., 2021). However, most evidence comes from research settings employing advanced neuroimaging techniques such as diffusion tensor imaging or morphometric analysis. To our knowledge, there are no published data from Eastern Europe describing structural brain abnormalities in CLWHIV based on routine clinical MRI. Importantly, HIV-related encephalopathy may present early in infancy, often before severe immunosuppression or other AIDS-defining conditions, and MRI changes such as WM lesions or cerebral atrophy may precede clinical signs of neurocognitive impairment (Mitchell, 2001).

This study aimed to describe the neurocognitive and neuroimaging status of CLWHIV in an observational cohort, and to explore associations between MRI findings and clinical and treatment history.

## Methods

The Republican Hospital for Infectious Diseases (RHID), St Petersburg, Russian Federation, is a specialist HIV center providing care for women and children in St Petersburg, and serves as a tertiary referral center for children from all regions of Russia.

In 2013, RHID introduced brain MRI and neurocognitive evaluation as part of routine care for CLWHIV aged ≤18 years at diagnosis of HIV and aged ≥6 years at the time of assessment visits (children are seen at least annually; in 2013 approximately 110 children met these criteria). Routine care data for all children and adolescents receiving HIV care were extracted from chart reviews as part of the RHID observational pediatric HIV cohort study. A cross-sectional analysis of the cohort data was conducted, using data on all children who received a brain MRI or neurocognitive function assessment between September 2013 and July 2015.

Participants underwent standard clinical brain MRI on a 1.5 Tesla Toshiba scanner. The imaging protocol included axial T1-weighted, T2-weighted, and fluid- attenuated inversion recovery (FLAIR) sequences, routinely employed in pediatric neuroimaging. Scans were independently reviewed by two experienced neuroradiologists (E.S. and A.M.d.A.), blinded to participants’ clinical and neuropsychological profiles. Assessment included the presence of focal WM lesions, diffuse WM lesions and global cerebral atrophy. For analysis purposes, scans were classified as abnormal when both reviewers agreed any of these features were present; discrepancies were resolved through consensus.

Neurocognitive function was assessed using the Russian version of the Wechsler Intelligence Scale for Children, 3^rd^ edition (WISC-III) (Filimonenko & Timofeev, 2006), administered by two experienced pediatric psychologists. The WISC-III comprises 15 subtests that generate Verbal IQ (VIQ) and Performance IQ (PIQ), which together yield the Full Scale IQ (FSIQ). The Verbal scale comprises Digit Span, Vocabulary, Similarities, Comprehension, Arithmetic and Information subtests; the Performance scale comprises Mazes, Block Design, Picture Arrangement, Picture Completion, Object Assembly and Coding subtests. Subtest scores are reported as scaled scores with a mean of 10 (standard deviation [SD] 3) (Wechsler, 1991); IQ indexes have a mean of 100 (SD 15).

From medical records, attending clinicians collected data on demographics and clinical history (sex, age at HIV diagnosis and ART initiation, current ART regimen, full CD4 history, AIDS diagnosis (including encephalopathy) ≥6 months prior to the assessment). Immunological and virological status was evaluated at time of assessment (closest CD4 cell count/CD4% and HIV RNA viral load to assessment date, within 12 months before to 3 months after assessment).

Immunosuppression for age at assessment (and any time prior) was categorized according to WHO definitions of none/not significant, mild, advanced and severe; for children aged >5 years this corresponds to CD4 cell counts of > 500, 350–499, 200–349 and < 200 cells/mm^3^, respectively (or a CD4% of < 15% for severe) (World Health Organization, 2007).

Participant characteristics were summarized descriptively. VIQ, PIQ, FSIQ and subtest scores were summarized as medians and IQRs due to the small sample size, together with the percentage scoring < 90 and < 80 (90 is the lower limit of the “average” categorization in WISC-III; 80–89 is considered low average, 70–79 borderline, ≤69 extremely low). For subtest scores, we considered the normal range to be 8–12 (Wechsler, 1991). MRI findings were summarized as the percentage of children with ≥ 1 abnormality recorded. Clinical characteristics of children with and without MRI abnormalities were compared using univariate logistic regression. We did not undertake adjusted analyses due to the small sample size. All analyses were conducted using Stata v17 (StataCorp, College Station, TX).

## Results and discussion

Thirty-seven CLWHIV were included in this analysis (Supplementary Table S1). 20/37 (54%) were female, median age at HIV diagnosis was 22 months [IQR 10–34] and at assessment (earlier of MRI or WISC) 10.5 years [IQR 8.7–11.9]. All but two had initiated ART, at a median age of 57 [IQR 27–93] months; median time since ART initiation was 4.6 [2.4–7.8] years. The ART regimen at the time of assessment was known for 22 children; all were on protease inhibitor-based regimens. All children underwent WISC-III assessment; 32 (89%) underwent MRI. Median time between WISC-III and MRI assessments was 3.3 months [IQR 0.3–11.3]; WISC-III was done before MRI in 11/ 32 (34%).

### WISC-III profiles

**Figure 1:**
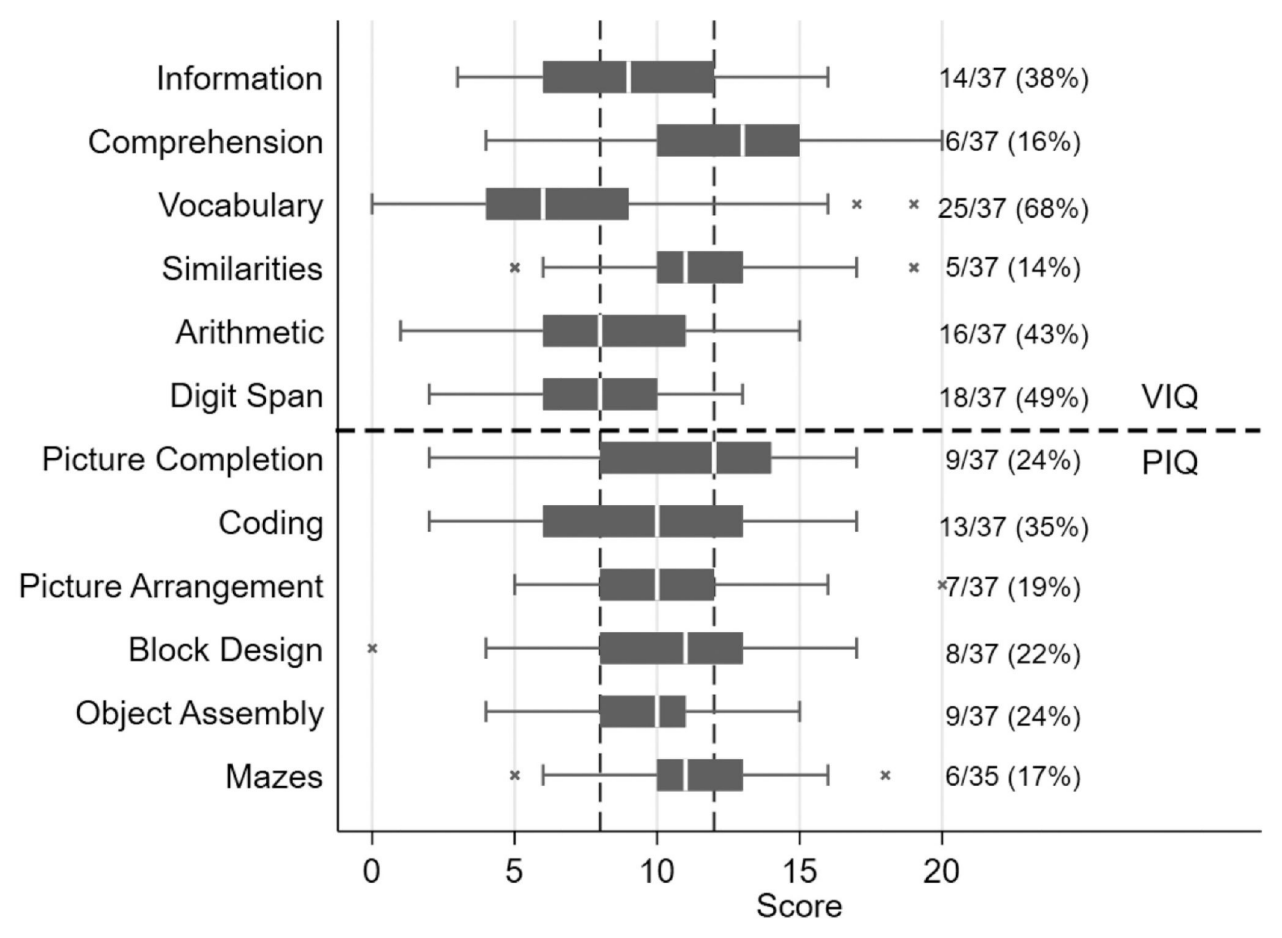
Distribution of WISC-III subtest scores (*n* = 37). Boxes show median and IQR; whiskers extend up to 1.5 × IQR. Vertical dashed lines indicate the normal range, 8-12; the number and percentage of children scoring <8 on each subtest is shown on the right. Sub-tests above the dashed line are components of the verbal IQ (VIQ), those below the dashed line are components of the performance IQ (PIQ).

Median WISC-III scores were 99 [IQR 91–111, range 62–123] for FSIQ, 97 [IQR 85–111, range 67–129] for VIQ and 101 [IQR 94–106, range 62–129] for PIQ (Supplementary Figure S1). Scores were below average (<90) in 9/37 (24%) for FSIQ, 5/37 (14%) for PIQ and 13/37 (35%) for VIQ, and < 80 (below the “low average”) in 5/37 (14%), 3/37 (8%) and 7/37 (19%), respectively. Median subtest scores were often within or above the normal range (Figure 1), but a large proportion of CLWHIV had scores which were below average, particularly for vocabulary (25/37 (68%)). Overall, 34/37 children (92%) scored below the normal range on at least one subtest; the median number of belowaverage subtest scores per child was 4 [IQR 1–5, range 0–10].

### MRI findings

Of the 32 participants who underwent standard brain MRI, 15 (46.9%) showed radiological abnormalities. Focal WM lesions were present in 12 children (37.5%), diffuse WM lesions in 4 children (12.5%), and cerebral atrophy in 3 children (9.4%). Three participants had both focal and diffuse WM lesions, one of whom also had cerebral atrophy. Detailed findings are given in Supplementary Table 2.

### Comparison of children with and without MRI abnormalities

In logistic regression analysis, older age at ART start was associated with MRI abnormalities (OR 1.37 (95% CI 1.01–1.86 per year increase, *p* = 0.05, *n* = 30 children with MRI assessments who had also started ART). Conversely, there was weak evidence that longer duration since ART initiation was associated with reduced odds of abnormalities (OR 0.76 (0.57–1.01) per year increase, *p* = 0.06, *n* = 30) (Table 1). There was no evidence of an association of MRI abnormalities with history of advanced/severe immunosuppression for age or maternal HIV (Table 1). Results were similar if the participant with history of encephalopathy was excluded. The association between history of AIDS diagnosis at time of assessment and MRI results could not be assessed due to small numbers.

In this study of neurocognitive function in CLWHIV in Russia, we found that, firstly, almost a quarter (24%) of participants scored below average on FSIQ with this driven mainly by low scores on VIQ rather than PIQ, although the median overall IQ scores were comparable to population norms. Secondly, almost half (47%) of CLWHIV had at least one abnormality on MRI, most commonly WM lesions. Third, older age at ART initiation was associated with higher odds of MRI abnormalities and conversely, longer duration since ART initiation was associated with lower odds of abnormalities.

Although the median WISC-III scores were within the normal range, 14–35% of children in this cross-sectional study scored below average on three key measures (VIQ, PIQ, FSIQ) of neurocognitive function. Our results are consistent with the broader literature in children living with HIV (Boivin et al., 1995; Koekkoek et al., 2008; Nichols, 2022; Nozyce et al., 2006). A study from Thailand, in which 87% of participants living with HIV (median age 8.9 years) had started ART a median of 35 weeks before assessment, noted low average VIQ scores, with PIQ less impacted (Puthanakit et al., 2010). In a Spanish study of adolescents, vocabulary was the most affected domain (García-Navarro et al., 2020). Expressive language deficits have often been reported, lasting into early adulthood (Redmond et al., 2016). Almost half of CLWHIV assessed had neuroimaging abnormalities, most commonly WM lesions, similar to a previous study from South Africa (Ackermann et al., 2014). Reasons for the neurocognitive impairments and MRI abnormalities may relate to prenatal HIV exposure, inflammation associated with HIV infection, co-infections, and/or socioeconomic factors (Bartlett et al., 2020). Various studies have explored cognitive interventions in CLWHIV and have reported mixed effects across different cognitive domains; the best approach to improve neurocognitive outcomes, other than early ART initiation, remains unclear (Thomas et al., 2020). In our setting, for CLWHIV with lower WISC-III scores or concerning MRI findings, follow-up recommendations were made, including referrals to neuropsychology, educational interventions, and therapies such as speech or occupational therapy.

In our univariate analysis, older age at ART start was associated with increased odds of MRI abnormalities. Participants in our study had started ART relatively late (at a median age of 57 months), and two were ART-naïve. Studies have shown that ART initiation in early infancy leads to better neurodevelopmental outcomes (Laughton et al., 2012). Improved access to early infant diagnosis from birth and ART initiation as soon as possible after diagnosis, as recommended by WHO, may prevent development of HIV-associated CNS impairment and MRI abnormalities.

Strengths of our study include detailed data on clinical history, detailed cognitive function assessments and MRI scans. Limitations include the small sample size and consequent univariate analysis, single-center cross-sectional design, lack of HIV-negative controls and lack of data on factors influencing cognitive outcomes such as maternal or child education, socioeconomic status and other social determinants of health. Thus we cannot conclude that the cognitive impairments and MRI abnormalities seen are caused by HIV. Nonetheless this study provides one of the first detailed assessments of cognitive function in CLWHIV in Eastern Europe. As a referral center, the study site may see a higher than average proportion of children with complex needs which may have led to overestimation of the prevalence of abnormalities and impairments. Approximately 30% of children in care in this center were included in this analysis; missing data was due to MRI machine breakdown and staff shortages, and would not be expected to bias the findings. Although volumetric or morphometric analyses were not conducted, the primary aim was to identify overt structural abnormalities detectable on conventional MRI. Visual assessments by two independent neuroradiologists provide a robust and clinically meaningful evaluation method, particularly relevant in resource-limited settings or routine clinical care. In future, longitudinal assessments would enable evaluation of changes in neurocognition and MRI findings over time and the effects of any supportive interventions. While updated versions of WISC are now available (Wechsler, 2014), WISC-III is the most recent version that is validated in the Russian language.

Scaled scores of 8–12 are considered average in the WISC-III manual (Wechsler, 1991), with 7 falling within an extended normative range. Thus, the prevalence of neurocognitive impairment may be slightly overestimated, although the Flynn effect suggests a trend toward higher population scores over time (Giangrande et al., 2022).

In summary, in this cross-sectional study, almost half of CLWHIV with MRI screening had at least one abnormality and 9 out of 10 had below average scores in at least one subtest of cognitive function. Earlier ART start was associated with reduced odds of MRI abnormalities. Our findings support efforts to ensure access to early HIV diagnosis and ART from early infancy, and CLWHIV with cognitive and neurological impairment may potentially benefit from supportive interventions to optimize their development.

## Supplementary Material

Supplemental data for this article can be accessed online at https://doi.org/10.1080/09297049.2025.2517150

Table 1

Table 2

## Figures and Tables

**Table 1:** Unadjusted odds ratios for relationship between participant characteristics and presence of MRI abnormalities (*n* = 32).

Characteristic	MRI abnormalities [n (%) or mean [SD], *n* = 15]	No MRI abnormalities [n (%) or mean [SD], *n* = 17]	Odds ratio (95% CI)	*p* value
Age at ART initiation (years)*^,†^	6 [3]	4 [2]	1.37 (1.01–1.86)	0.05
Time since ART start (years)*^, †^	4 [3]	6 [3]	0.76 (0.57–1.01)	0.06
History of AIDS diagnosis *(n* = 29)	0 (0)	3 (18)	NA	NA
Maternal HIV	8 (53)	12 (71)	0.48 (0.11–2.04)	0.32
Current/previous advanced or severe immunosuppression (*n* = 31)	6 (40)	7 (41)	0.86 (0.21–3.58)	0.83
Age at MRI (years)*	10.3 [2.3]	10.5 [2.7]	0.97 (0.73–1.28)	0.82
On ART before MRI	14 (93)	16 (94)	0.88 (0.05–15.33)	0.93

* OR per year increase.

† Amongst 30 who had started ART before MRI.

NA = not applicable.

MRI, magnetic resonance imaging; CI, confidence interval; ART, antiretroviral therapy; AIDS, acquired immunodeficiency syndrome.
