# Supplementary material for: Brain MRI and neurocognitive characteristics of children and adolescents living with HIV
Source: Child Neuropsychol. Author manuscript; Available in PMC 2026 Jun 4. (PMC7619127; doi:10.1080/09297049.2025.2517150)
Supplement: Table 1 [file EMS213955-supplement-Table_1.docx]

**Supplementary Table 1: Characteristics of children who received an MRI or neurocognitive assessment (n = 37)**

| **Characteristic** | | **Median [IQR] or n (%)** |
| --- | --- | --- |
| Sex, female (n = 37) | | 20 (54) |
| Preterm birth (<37 completed weeks) (n = 37) | | 2 (5) |
| Living in an institution (n = 37) | | 5 (14) |
| Maternal HIV (n = 36) | | 23 (64) |
| Age at HIV diagnosis (months) (n = 37) | | 22 [10 - 34] |
| Age at ART initiation (months) (n = 35)* | | 57 [27 - 93] |
| ***At time of earliest assessment (MRI or WISC-III)*** | | |
| Age (years) (n = 37) | | 10.5 [8.7 - 11.9] |
| Immunosuppression category  (n = 35) | None / not significant | 29 (83) |
|  | Mild | 3 (9) |
|  | Advanced | 1 (3) |
|  | Severe | 2 (6) |
| Current / previous advanced or severe immunosuppression for age  (n = 36) | | 15 (42) |
| History of AIDS diagnosis (n = 37)** | | 3 (8) |
| History of encephalopathy diagnosis (n = 37) | | 1 (3) |
| CD4 cell count (cells/mm^3^) (n = 36) | | 730 [573 - 1081] |
| Nadir CD4 cell count (cells/mm^3^) (n = 36) | | 522 [367 – 699] |
| Undetectable viral load (<150 copies/mL) (n = 32) | | 24 (75) |
| Time since ART initiation (years) (n = 35)* | | 4.6 [2.4 - 7.8] |

* Amongst children starting ART before first assessment

** At least six months before first assessment. Three additional children had later AIDS diagnoses (one HIV wasting syndrome diagnosed 1 day before assessment and two cases of encephalopathy diagnosed 6 and 67 days later)

IQR, interquartile range; HIV, human immunodeficiency virus; ART, antiretroviral therapy; MRI, magnetic resonance imaging; WISC, Wechsler intelligence scale for children; AIDS, acquired immunodeficiency syndrome
