# Supplementary material for: Brain MRI and neurocognitive characteristics of children and adolescents living with HIV
Source: Child Neuropsychol. Author manuscript; Available in PMC 2026 Jun 4. (PMC7619127; doi:10.1080/09297049.2025.2517150)
Supplement: Table 2 [file EMS213955-supplement-Table_2.docx]

**Supplementary Table 2.** Detailed summary of brain MRI findings among 32 children and adolescents living with HIV. The table shows the presence (1) or absence (0) of focal white matter (WM) lesions, diffuse WM lesions, and cerebral atrophy for each participant. Additional extracerebral or incidental findings are listed in the final column.

| **Patient** | **Focal WM Lesions** | **Diffuse WM Lesions** | **Atrophy** | **Other MRI Findings** |
| --- | --- | --- | --- | --- |
| **1** | 0 | 0 | 0 |  |
| **2** | 0 | 0 | 0 |  |
| **3** | 1 | 1 | 0 | Sphenoidal sinus occupation |
| **4** | 1 | 0 | 0 | Chiari malformation |
| **5** | 0 | 0 | 0 | Right mastoid occupation |
| **6** | 1 | 0 | 1 | Subcortical focal lesion |
| **7** | 1 | 0 | 0 |  |
| **8** | 1 | 0 | 0 | Hypothalamic lipoma |
| **9** | 1 | 0 | 0 |  |
| **10** | 0 | 0 | 0 | Cervical adenopathies |
| **11** | 0 | 0 | 0 | Maxillary retention cyst |
| **12** | 0 | 0 | 0 |  |
| **13** | 0 | 0 | 0 |  |
| **14** | 1 | 0 | 0 |  |
| **15** | 1 | 0 | 0 | Paranasal sinus thickening |
| **16** | 0 | 0 | 0 | Mastoid occupation |
| **17** | 1 | 0 | 0 |  |
| **18** | 0 | 0 | 0 |  |
| **19** | 1 | 1 | 0 | Perivascular dilations |
| **20** | 0 | 0 | 0 |  |
| **21** | 0 | 0 | 0 |  |
| **22** | 0 | 0 | 0 | Paranasal sinus thickening |
| **23** | 0 | 0 | 0 |  |
| **24** | 0 | 0 | 0 | Cervical adenopathies |
| **25** | 0 | 0 | 0 |  |
| **26** | 0 | 1 | 0 |  |
| **27** | 1 | 0 | 0 |  |
| **28** | 0 | 0 | 0 |  |
| **29** | 0 | 0 | 1 |  |
| **30** | 1 | 1 | 0 | Cervical adenopathies |
| **31** | 0 | 0 | 1 |  |
| **32** | 0 | 0 | 0 |  |
